# The Human Microbiota and Prostate Cancer: Friend or Foe?

**DOI:** 10.3390/cancers11040459

**Published:** 2019-03-31

**Authors:** Francesco Massari, Veronica Mollica, Vincenzo Di Nunno, Lidia Gatto, Matteo Santoni, Marina Scarpelli, Alessia Cimadamore, Antonio Lopez-Beltran, Liang Cheng, Nicola Battelli, Rodolfo Montironi, Giovanni Brandi

**Affiliations:** 1 Division of Oncology, S. Orsola-Malpighi Hospital, 40138 Bologna, Italy; veronica.mollica7@gmail.com (V.M.); dinunnovincenzo88@gmail.com (V.D.N.); lidia.gatto83@gmail.com (L.G.); 2 Macerata Hospital, 62100 Macerata, Italy; mattymo@alice.it (M.S.); nicola.battelli@sanita.marche.it (N.B.); 3 Section of Pathological Anatomy, Polytechnic University of the Marche Region, School of Medicine, United Hospitals, 60126 Ancona, Italy; m.scarpelli@univpm.it (M.S.); alessiacimadamore@gmail.com (A.C.); r.montironi@univpm.it (R.M.); 4 Department of Surgery, Cordoba University Medical School, 14004 Cordoba, Spain; em1lobea@gmail.com; 5 Department of Pathology and Laboratory Medicine, Indiana University School of Medicine, Indianapolis, IN 46202, USA; liang_cheng@yahoo.com; 6 Oncology Unit, Department of Experimental, Diagnostic and Specialty Medicine, Sant’Orsola-Malpighi Hospital, University of Bologna, 40138 Bologna, Italy; giovanni.brandi@unibo.it

**Keywords:** microbiota, microbiome, prostate cancer, genitourinary cancers

## Abstract

The human microbiome is gaining increasing attention in the medical community, as knowledge on its role not only in health but also in disease development and response to therapies is expanding. Furthermore, the connection between the microbiota and cancer, especially the link between the gut microbiota and gastrointestinal tumors, is becoming clearer. The interaction between the microbiota and the response to chemotherapies and, more recently, to immunotherapy has been widely studied, and a connection between a peculiar type of microbiota and a better response to these therapies and a different incidence in toxicities has been hypothesized. As knowledge on the gut microbiota increases, interest in the residing microbial population in other systems of our body is also increasing. Consequently, the urinary microbiota is under evaluation for its possible implications in genitourinary diseases, including cancer. Prostate cancer is the most common cancer in the male population; thus, research regarding its etiology and possible factors correlated to disease progression or the response to specific therapies is thriving. This review has the purpose to recollect the current knowledge on the relationship between the human microbiota and prostate cancer.

## 1. Introduction

The multifactorial process of tumorigenesis and the mechanisms promoting cancer progression or response to therapies are everlasting areas of interest for oncologists. In this scenario, the role of the human microbiota and microbiome (i.e., the whole gastrointestinal bacterial population and relative genomes) is gaining attention, as increasing evidence links these commensal bacteria not only to the maintenance of health but also to the development of multiple diseases, including cancer [1,2,3]. The human microbiota can be defined as the microorganisms, such as bacteria, archaea, fungi, and protozoa that physiologically live in the epithelial barrier surfaces of our body [4]. The microbiome represents the totality of microbes and their genetic information [5].

The host and its microbiota usually are in symbiotic equilibrium, which, when altered by several stressors such as environmental factors, dietary changes, or drugs, can lead to a dysbiosis that in turn can promote many diseases [6].

Knowledge of the composition of the human microbiota is rapidly increasing through 16S ribosomal RNA or DNA sequencing metagenomics approaches that either provide strong information about taxonomy, thus simplifying the complexity of bacterial classification, or whole bacterial genome, unravelling several bacterial functions. Thus, since information on the normal composition and functions of the microbiota in the gastrointestinal system and the genitourinary system has already been gained, attention is now focused on the alteration in its composition when specific diseases arise, including cancer [2].

Furthermore, in the oncologic field a particular interest is in studying the role of the microbiota connected with specific therapies, including chemotherapy and immunotherapy [7,8].

Among malignancies, prostate cancer is the most frequent in the male population, with 175,000 estimated new cases and about 32,000 estimated deaths in the United States of America in 2019 [9]. The pathogenesis of this type of cancer is mainly linked to its dependence on androgen hormones. Accordingly, the main treatments of prostate cancer are based on anti-androgen therapies. The discovery of other pathogenic events, possible risk factors, or the mechanisms behind the eventual state of resistance to therapies are of great interest.

In this still immature scenario, with increasing interest in the microbiota as a new player in several human diseases, we present a review of the connections between the microbiota and cancer with a specific focus on prostate cancer.

## 2. The Human Microbiota

The role of the microbiota has been gaining more attention as knowledge of its functions has grown: it is implicated in metabolism, neurological and cognitive functions, hematopoiesis, inflammation, and immunity [1,10].

The composition of the microbiota varies depending on genetic factors, colonization at time of birth, type of delivery, host’s lifestyle, exposure to antibiotics or other drugs, dietary factors, and diseases [11,12,13,14,15].

Moreover, the microbiota composition changes according to the system environment in which it resides. The intestinal microbiota is mainly composed of five bacterial phyla: *Firmicutes*, *Bacteroidetes*, *Actinobacteria*, *Proteobacteria*, and *Verrucomicrobia* [16,17,18]. The most represented anaerobes are *Bacteroides*, *Eubacteria*, *Bifidobacteria*, *Clostridia*, *Peptostreptococci*, and *Ruminococci* [16].

For a long time, the urinary tract was considered to be a sterile environment, but recent discoveries gained through PCR and 16S ribosomal RNA sequencing technology on urine samples have proved that it contains a peculiar microbiota [19,20,21]. The urinary microbiota composition seems to differ according to gender, as a result of the anatomical structure and hormonal differences between the sexes, and of age [21]. The genera *Lactobacillus* and *Gardnerella* are predominant in the female microbiota, whereas the male microbiota presents a higher percentage of *Corynebacterium*, *Staphylococcus*, and *Streptococcus* [22,23].

The microbiota differs from healthy people and patients with different urinary diseases. Lewis et al. analyzed clean-catch midstream urine of healthy individuals and found *Jonquetella*, *Parvimonas*, *Proteiniphilum*, and *Saccharofermentans* to be the more represented genera, with a more heterogeneous mix of bacterial genera in female samples, which also presented members of the phyla *Actinobacteria* and *Bacteroidetes*, which were absent from the male samples [21]. Willner and colleagues characterized the bacteria present in 50 patients with acute uncomplicated urinary tract infections using culture-independent sequence-based methods and reported that the predominant taxa were *Escherichia (Escherichia coli* being the most common organism in general), *Anaerococcus*, *Peptoniphilus*, *Streptococcus*, *Lactobacillus*, *Staphylococcus*, and *Pseudomonas* [24]. Pearce et al. sequenced urine samples collected through transurethral catheter from healthy women and women with urgency urinary incontinence (UUI) [25]. The results showed two different microbiomes between the two cohorts: the patients with UUI presented a higher representation of nine genera: *Actinobaculum*, *Actinomyces*, *Aerococcus*, *Arthrobacter*, *Corynebacterium*, *Gardnerella*, *Oligella*, *Staphylococcus*, *Streptococcus*, and a decreased percentage of *Lactobacillus* [25].

The role of the urogenital microbiota is under evaluation for its possible implications in urogenital diseases, both benign, such as urinary tract infections, urinary incontinence, interstitial cystitis, chronic prostatitis, urolithiasis [19,26,27], and malign, in regards to bladder, prostate, and kidney cancer [28,29].

The host and the microbiota share a complex balanced relationship that can be overthrown in a state of dysbiosis consequent to environmental changes that alter the microbiome or the host, leading to promotion of diseases [6]. The homeostasis is based on the integrity of the epithelial barrier colonized by the microbiota and that protects the host. An alteration in the composition of the microbiota, known as dysbiosis, can cause a breach of the epithelial barrier and can result in inflammatory bowel diseases, allergies, metabolic disorders, and cancer [30,31,32,33].

The gut microbiota is gaining a relevant role in the tumorigenesis process: increasing evidence suggests that the intestinal microbiota may have both an anti-tumoral [34,35,36] and a pro-tumoral effect [37,38,39,40]. The microbiota can influence cancer development and progression because it seems to be able to modulate inflammation and genomic stability of host cells [6,41].

Recently, an unexpected presence of bacteria within tumor tissue, both in malignancies of gastrointestinal tract or outside the gut, has been found, and these bacteria seem able to modulate response both to chemotherapy and immunotherapy [7,42,43,44,45,46]. Actually, *Fusobacterium nucleatum*, which has been detected both in primary colorectal cancer and relative liver metastases, is associated with a worse prognosis compared to germ-free tumors [47]. Similarly, in pancreatic cancer, a large bacterial population suppresses monocytic differentiation, thus inducing T-cell anergy and interfering with response to gemcitabine [48].

Furthermore, the microbiota has been implicated in response to several chemotherapies, like 5-fluorouracil, cyclophosphamide, irinotecan, oxaliplatin, gemcitabine, and methotrexate [49,50,51,52,53,54,55].

## 3. Microbiota and Prostate Cancer

Prostate cancer is the second leading cause of death and the first type of cancer for incidence in the male population [9]. Its pathogenesis and natural history are strongly linked to its dependence on androgen hormones, but the discovery of other risk factors that may participate in cancer development, progression, or resistance to therapies is of particular interest. Risk factors evaluated for their potential implication in the etiology of prostate cancer are viral and bacterial infections, inflammatory stimuli, and environmental factors, like diet and lifestyle [56,57,58].

The evidence available in the literature is pointing out that the human microbiome residing in multiple anatomic sites, such as urinary tract, gastrointestinal tract, and oral cavity, may play an important role in prostate health and diseases like prostatitis, chronic pelvic pain syndrome, benign prostatic hyperplasia, and prostate cancer [59].

The association between infectious disease, inflammation, and cancer has been widely studied in many malignancies. There is increasing knowledge about the role of the microbiota in promoting the status of chronic inflammation and its possible implication in prostate cancer development [60]. The discovery of a urinary microbiome composed of many different microorganisms supports this hypothesis because the prostate is exposed to many inflammatory stimuli deriving from the bacteria of this environment. In fact, the anatomy of the prostate, which is in close proximity with the urethra, exposes this organ to the microorganism residing in the urinary tract. Shrestha and colleagues recently carried out a study on urine samples from men prior to undergoing prostate biopsy in order to determine whether the urinary microbiome could be associated with the presence of cancer, cancer grade, and the type and degree of prostate inflammation [23]. The study demonstrated that men with biopsy-proven prostate cancer presented a higher proportion of a cluster of bacteria frequently associated with urogenital infections, like prostatitis, bacterial vaginosis, and urinary tract infections, than biopsy-negative samples. This cluster was characterized by *Streptococcus anginosus*, *Anaerococcus lactolyticus*, *Anaerococcus obesiensis*, *Actinobaculum schaalii*, *Varibaculum cambriense*, and *Propionimicrobium lymphophilum*. Of note, some species were differently represented in the presence or absence of acute inflammation or in high- versus low-grade cancers.

Moreover, inflammation has been associated with development of prostate cancer though other different putative mechanisms [60]. Prostate inflammation, which is very common in adult men, is characterized by an increased number of inflammatory cells into the prostate tissue that have been hypothesized to be correlated with cancer development and progression [57,61]. For example, strong tumor infiltrating lymphocytes expression has been associated with short PSA-free survival in patients with local prostate carcinoma treated with prostatectomy [62]. Furthermore, it has been speculated that inflammation could promote cancer development through the release of reactive oxygen species (ROS) and reactive nitrogen species by immune cells that could directly damage DNA and cause genetic instability [57]. The oxidative stress and the consequent cellular damage and death are supposed to stimulate proliferation of atrophic luminal epithelial cells that create the regions known as proliferative inflammatory atrophy (PIA), characterized by areas of glandular atrophy and epithelial cell proliferation associated with chronic inflammation [63]. PIA is a regenerative lesion characterized by elevated Bcl-2 expression and consequent low apoptotic rate [63]. These lesions have been identified as precursors of prostate malignancies as they have been observed in direct morphological transition with high-grade prostate intraepithelial neoplasia (PIN), a pre-neoplastic lesion, and even with adenocarcinoma [63,64,65].

Chronic inflammation is known to be associated with development of multiple types of cancer: among the mechanisms behind this process there is the production of reactive chemical compounds, like superoxide, hydrogen peroxide, and nitric oxide released from cells of the immune system activated during chronic inflammation, which cause oxidative and nitrosative damage to DNA in the epithelial cells [66]. This process produces cell death and stimulates regeneration of epithelial cells exposed to DNA-damaging agents, thus resulting in an increased risk of mutation [57].

**Figure 1:**
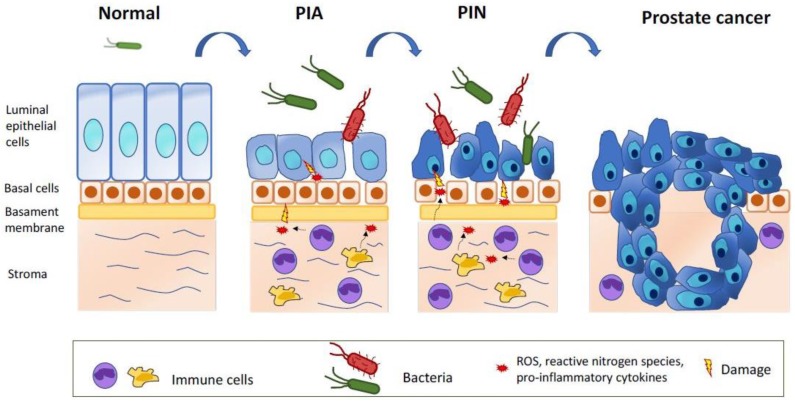
Inflammation and prostate cancer development stages: the healthy prostate contains in the prostatic fluid antimicrobial peptides and no or very few commensal microorganisms, which are not pathological when the integrity of luminal epithelial barrier is maintained.

The main causes of prostate inflammation are infections like bacterial prostatitis frequently linked to *E. Coli* or other species of *Enterobacteriaceae* [67], hormonal alterations such as estrogen exposure that could cause architectural alterations, physical trauma consequent to corpora amylacea, urine reflux, and diets rich in carcinogens that can reach the prostate and cause DNA damage [57]. The connection between inflammation and prostate cancer development stages is represented in Figure 1.

In a pathological state, for example, prostate infection or physical trauma due to corpora amylacea or urine reflux, the outgrowth of pathogenic bacteria and the breach of the epithelial barrier can induce an inflammatory state, characterized by an infiltration of immune cells (macrophages, neutrophils, and lymphocytes), which release reactive oxygen species (ROS), reactive nitrogen species, and pro-inflammatory cytokines, causing DNA damage, cell injury, and cell death. The resulting chronic inflammation state stimulates epithelial cell regeneration, creating primarily the regions known as proliferative inflammatory atrophy (PIA), which further evolve into low-grade and high-grade prostate intraepithelial neoplasia (PIN) and, finally, into prostate adenocarcinoma.

Liss et al. analyzed using 16S rRNA amplicon sequencing the rectal microbiome profile from rectal swab of patients undergoing transrectal prostate biopsy. No differences were found among men diagnosed with and without prostate cancer, except for an enrichment of the proinflammatory species *Bacteroides* and *Streptococcus* in patients with prostate cancer [68]. Furthermore, the authors tried to identify a possible microbiome profile that could predict prostate cancer risk on the basis of ten aberrant metabolic pathways. They reported that bacteria associated with carbohydrate metabolism pathways were in abundance in patients with prostate cancer, whereas bacteria associated with folate, biotin, and riboflavin were less abundant. Even though the search for specific microbiome profiles with diagnostic value is undoubtedly interesting, it may be too soon to accomplish this task based on the available data, and larger metatranscriptomic and metabolomics studies are warranted [69].

The microbial ecosystem of tumoral, peritumoral, and non-tumoral prostate tissue collected after radical prostatectomy has been analyzed through massive ultradeep pyrosequencing. In all types of samples, the dominant phylum was *Actinobacteria*, of which the most abundant genera were *Propionibacterium* in all the three different tissues, followed by *Firmicutes* and *Proteobacteria*; *Staphylococcus* spp. were more represented in the tumor and peri-tumor tissues [70].

With regards to the role of gastrointestinal microbiome in cancer treatment response, the evidence on this connection in prostate cancer incomplete, unlike other types of tumors, as previously reported. Sfanos and colleagues profiled the fecal microbiota of 30 subjects using 16S rDNA amplicon sequencing, including healthy male volunteers and men with localized, biochemically recurrent, and metastatic prostate cancer [71]. The results showed a greater abundance of *Akkermansia muciniphila* and *Ruminococcaceae* spp. in the gastrointestinal microbiota of men being treated with oral androgen receptor axis-targeted therapies such as bicalutamide, enzalutamide, and abiraterone acetate. These species are also linked to response to anti-PD-1 immunotherapy [43,44,72,73]. Immune checkpoint inhibitors are currently under evaluation in patients with prostate cancer [74]; thus, the connection between the microbiota and the response to these therapies is an interesting area of research in order to identify putative modulating factors of efficacy of immunotherapy. Furthermore, in the study by Sfanos et al., the microbiota composition of men being treated with anti-androgens was different than the one of men treated only with gonadotropin-releasing hormone agonists/antagonists or not being treated, since it was enriched with functional pathways involving steroid biosynthesis [71]. The authors speculate that there could be a deep connection between microbiota and steroidogenesis, in particular, that species of bacteria capable of steroid biosynthesis could create different pathways of androgen production, thus interfering with the response to anti-androgen therapies. The interaction between bacteria and steroidogenesis has been previously investigated, and there is evidence that some bacteria are able to metabolize and catabolize estrogen and androgen precursors, thus affecting their systemic levels [75,76]. There is further evidence in support of the connection between the gut microbiota and hormones: the gut microbiota produces, secretes, and regulates levels of hormones, affecting host metabolism, immunity, and behavior [77]. This relationship, though, seems to be bidirectional as the microbiota is itself affected by host’s hormones: in fact, host’s factors like diet, exercise, mood, general health state, stress, and gender can alter hormonal levels, which can produce an alteration in the microbiota in terms of bacterial growth or increased or decreased virulence [77]. Furthermore, studies on animal models suggest that the gastrointestinal microbiota may be affected also by androgen deficiency as in a castration state: there is evidence showing an increased *Firmicutes*/*Bacteroidetes* ratio and *Lactobacillus* species in the feces of high-fat diet-fed castrated mice, and this alteration in the microbiota composition was associated with abdominal obesity [78]. This intricate connection between microbiota and hormones is of particular interest, especially regarding those cancers deeply influenced by hormones levels, such as prostate cancer, that recognize androgen hormones as the main factor guiding its development, treatment, and resistance to therapies [79]. Moreover, there is evidence of the involvement of the microbiome also in breast cancer, another type of tumor strongly dependent on hormones, especially through the interaction of the microbiome and estrogen metabolism [80].

The studies investigating the connection between microbiota and prostate cancer previously discussed are summarized in Table 1.

## 4. Conclusions

The deep interaction of the human microbiota and its host seems to be fundamental in maintaining a balance that, if altered, could lead to many diseases. The knowledge of the mechanisms underlying cancer development or the instauration of resistance to therapies is essential in order to overcome them. The bacteria resident in the human body seem to influence many steps of the natural history of cancers. 

The microbiota and its environment live in an in intertwined relationship, and the challenge seems to be to understand which is the first to influence the other. With the increasing evidence available, it can be speculated that, while on one hand the microbiota could be a promoter in the development or progression of cancer, on the other hand it also seems possible that cancer itself could change the microenvironment, thus causing changes in the microbiota composition. Moreover, the microbiota is a double-sided element for its host: friend when in balance and foe if a state of dysbiosis occurs. It can also modulate drug activity, acting as a sort of pharmacist inside our body.

In prostate cancer, the role of the microbiota is still not well understood, but increasing evidence supports its putative role in health and disease of the prostate. The scarce and still mostly speculative nature of the body of work investigating the association of microbiome and prostate cancer requires a deeper understanding of this subject through more extensive studies on the possible implications of the microbiota in various aspects of prostate cancer.

## Figures and Tables

**Table 1:** Studies discussed in the manuscript investigating the connection between microbiota and prostate cancer.

Reference	Samples	Findings	Bacteria
Shrestha E et al. *J. Urol.* 2018 [23]	Urine samples from men prior to undergoing prostate biopsy	Men with biopsy proven prostate cancer presented a higher proportion of a cluster of bacteria frequently associated with urogenital infections, like prostatitis, bacterial vaginosis, and urinary tract infections	*Streptococcus anginosus*, *Anaerococcus lactolyticus*, *Anaerococcus obesiensis*, *Actinobaculum schaalii*, *Varibaculum cambriense*, *Propionimicrobium lymphophilum*
Liss MA et al. *Eur. Urol.* 2018 [68]	Rectal swab of patients undergoing transrectal prostate biopsy	Enrichment of the proinflammatory species *Bacteroides* and *Streptococcus* in patients with prostate cancer. Bacteria associated with carbohydrate metabolism pathways were in abundance in patients with prostate cancer, whereas bacteria associated with folate, biotin, and riboflavin were less abundant.	*Bacteroides*, *Streptococcus*
Cavarretta I et al. *Eur. Urol.* 2017 [70]	Tumoral, peritumoral, and non-tumoral prostate tissue collected after radical prostatectomy	In all types of samples, the dominant phylum was *Actinobacteria* (most abundant genera: *Propionibacterium*), followed by *Firmicutes* and *Proteobacteria*; *Staphylococcus* spp. were more represented in the tumor and peri-tumor tissues.	*Actinobacteria, Firmicutes*, *Proteobacteria*
Sfanos KS et al. *Prostate Cancer and Prostatic Diseases* 2018 [71]	Fecal samples of healthy male volunteers and men with localized, biochemically recurrent and metastatic prostate cancer	Greater abundance of *Akkermansia muciniphila* and *Ruminococcaceae* spp. in the gastrointestinal microbiota of men on treatment with oral androgen receptor axis-targeted therapies such as bicalutamide, enzalutamide, and abiraterone acetate	*Akkermansia muciniphila*, *Ruminococcaceae* spp
